# The use of fermented buckwheat to produce l-carnitine enriched oyster mushroom

**DOI:** 10.1186/s13568-018-0664-6

**Published:** 2018-08-27

**Authors:** Tae-kyung Lee, Thi Thanh Hanh Nguyen, Namhyeon Park, So-Hyung Kwak, Jeesoo Kim, Shina Jin, Gyu-Min Son, Jaewon Hur, Jong-In Choi, Doman Kim

**Affiliations:** 1 Graduate School of International Agricultural Technology and Center for Food and Bioconvergence, Seoul National University, Pyeongchang, 25354 South Korea; 2 The Institute of Food Industrialization, Institutes of Green Bio Science &Technology, Seoul National University, Pyeongchang, 25354 South Korea; 3 Mushroom Research Institute, GARES, Gwang-Ju, Gyeonggi 12805 South Korea

**Keywords:** Antioxidant, Cell cytotoxicity, Functional food, Phenolic compounds, Oyster mushroom, Buckwheat

## Abstract

l-Carnitine is an essential compound that shuttles long chain fatty acids into mitochondria. The objective of this study was to produce l-carnitine enriched oyster mushroom (*Pleurotus ostreatus*) using common buckwheat fermented by *Rhizopus oligosporus.* Mushroom grown on common buckwheat medium contained 9.9–23.9% higher l-carnitine (186.3 mg/kg) than those grown on basal medium without any buckwheat addition. Those grown on fermented common buckwheat medium contained the highest l-carnitine content (201.2 mg/kg). Size index and lightness of mushroom pileus (L*) were also the highest (100.7 and 50.6, respectively) for those grown in medium added with fermented common buckwheat (20%, w/w). Antioxidant activities of both mushroom extracts (1.5 mg/mL) showed the same level as 38.7% for mushroom grown in media added with common buckwheat or fermented common buckwheat. At the treatment concentration of 300 μg/mL, viabilities of murine macrophage cell line Raw 264.7 cells treated with ethanol extract of oyster mushroom grown on buckwheat medium ranged from 58.9 to 67.8%. The oyster mushroom grown on buckwheat and fermented buckwheat medium can be used as one of the substitutes for meat based diets.

**Electronic supplementary material:**

The online version of this article (10.1186/s13568-018-0664-6) contains supplementary material, which is available to authorized users.

## Introduction

Mushroom has been used as traditional foods and medicine in eastern Asia due to its functional properties. World production of mushroom has been grown rapidly since late 20th century. China accounted for over 70% of world production of mushroom in 2016 (FAOSTAT 2016). *Pleurotus ostreatus,* the second most cultivated mushroom in the world, is commonly known as “Oyster mushroom” and “Hiratake” (Sánchez 2010). Oyster mushroom has various biological functions, including antimicrobial activity against *Escherichia coli,* and *Staphylococcus aureus* (Akyuz et al. 2010), antineoplastic activity against Ehrlich ascetic tumor (Wolff et al. 2008), antioxidant activity (Venkatakrishnan et al. 2009), antitumor activity of *P. ostreatus* mycelia-derived proteoglycans (Sarangi et al. 2006), and immunomodulatory activity of pleuran, an insoluble polysaccharide extracted from *P. ostreatus* (Jesenak et al. 2013). Yields and chemical composition of mushroom are enhanced by adding essential elements such as selenium (da Silva et al. 2012; Kristensen et al. 2012; Vieira et al. 2013).

Among buckwheat species, common buckwheat (*Fagopyrum esculentum*) and Tartary buckwheat (*Fagopyrum tataricum*) are cultivated for human food. Buckwheat (*Fagopyrum* spp.) is a good source of nutritionally valuable amino acids, dietary fibers, and minerals such as zinc and copper (Bonafaccia et al. 2003; Zhang et al. 2012). In addition, buckwheat has been found to contain flavonoids, fagopyrin, tocopherols and phenolic substances such as 3-flavanols, rutin, phenolic acids, and their derivatives with antioxidant activity (Fabjan et al. 2003; Jiang et al. 2007). Also, buckwheat has higher content of amino acids such as methionine and lysine (precursors of l-carnitine) than rice and other pseudo cereals (Bonafaccia et al. 2003; Mota et al. 2016). l-Carnitine (β-hydroxy-γ-*N*-trimethylaminobutyric acid) is a non-essential amino acid derivative and natural compound occurring most in red meat (Demarquoy et al. 2004). Its major role is a carrier of long chain fatty acid into mitochondria for beta-oxidation. l-carnitine is considered as a weight-loss product because of its function related to fat metabolism. Clinical studies have shown that regular l-carnitine intake can lead to weight loss in human (Novakova et al. 2016). Although l-carnitine is synthesized from essential amino acids, lysine and methionine in human, 75% of l-carnitine is exogenously obtained from food, especially meat and milk (Steiber et al. 2004). However, there have been many controversies regarding meat based diets for human health issue (Chao et al. 2005; Pan et al. 2011; Wang and Beydoun 2009), since most l-carnitine is supplied from meat based diet, vegetarians have to eat more plants to have enough intake of l-carnitine (Cave et al. 2008; De Vivo and Tein 1990). Buckwheat without additional nutrients has been fermented using *Rhizopus oligosporus* (*R. oligosporus*), producing four times higher amount of l-carnitine than original buckwheat (Park et al. 2017). The fermented buckwheat extract powder with increased amounts of l-carnitine was used as a complex additive in poultry feed to Hy-Line brown hens and resulted higher egg production and quality than the control group, and increased the l-carnitine content in the yolk (Park et al. 2017). Therefore, in this study, we focus on the utilization of buckwheat and fermented buckwheat as medium materials to improve the biological activity of oyster mushroom. In addition, the morphological characteristics, l-carnitine content, antioxidant properties, and cell cytotoxicity of oyster mushroom were investigated.

## Materials and methods

### Microorganisms and culture condition

*Rhizopus microspores* var. *oligosporus* (*R. oligosporus*) was obtained from our previous study (Park et al. 2017) and deposited as KCCM 11948P (Korean Culture Center of Microorganisms, Seoul, Korea). *R. oligosporus* was maintained on potato dextrose agar (PDA) (Difco, USA) and incubated at 28 °C until spore formation (Park et al. 2017). *P. ostreatus* was obtained from Mushroom Research Institute (Gwangju, Gyeonggi, Korea) (Choi et al. 2013).

### Preparation of fermented buckwheat

Tartary buckwheat and common buckwheat were purchased from Bongpyeong (Pyeongchang bongpyeong memil, Gangwon-do, Korea). Fermented buckwheat was prepared with a modified method described previously (Park et al. 2017). Briefly, unhulled buckwheat (750 g) was soaked in water (1.5 L) for 6 h in a metal tray (height × width × length = 5.5 cm × 15.5 cm × 28.5 cm) and sterilized at 121 °C for 25 min. Then 7.5 mL of *R. oligosporus* spore solution (1 × 10^6^ spores/mL) was inoculated into each sterilized buckwheat tray after cooling to room temperature followed by incubation at 30 °C until mycelia covered the surface of tray (76 h) with relative humidity maintained above 90%. Fermented buckwheat was lyophilized at − 10 to 0 °C under 10 Pa for 4 days (Eyela, Tokyo, Japan). It was then milled with a blender (Hanil, Seoul, Korea) and stored at − 20 °C blocking light to preserve sensitive compounds such as quercetin and rutin for further analysis.

### Analyses of fermented buckwheat

Total phenolic content (TPC) of buckwheat was measured with Folin-Ciocalteu method (Singleton et al. 1999). Buckwheat and fermented buckwheat were suspended in 70% ethanol at 50 mg/mL and the supernatant was separated by centrifugation at 13,500×*g* for 10 min. The supernatant, diluted fivefold with water (120 µL) was then mixed with 15 µL of Folin-Ciocalteu reagent for 3 min on a microplate shaker (300 rpm, Thermo Fisher Scientific, Waltham, MA, USA). Then 15 µL of 10% (w/v) Na_2_CO_3_ was added to the mixture followed by shaking for 30 min. TPC was determined at wavelength of 760 nm with a microplate reader (Molecular Devices, Sunnyvale, CA, USA) using gallic acid as standard (10 to 100 µg/mL). Total flavonoids content (TFC) of the extract was measured using aluminum chloride method (Chang et al. 2002). The supernatant, diluted fivefold with methanol (2 mL) was then mixed with 10% (w/v) AlCl_3_ (100 µL) dissolved in water and 0.1 mM Potassium acetate (100 µL). TFC was then determined at wavelength of 415 nm using quercetin as the standard (10 to 100 µg/mL).

### Mushroom spawning and fruiting in field scale

Cultivation of *P. ostreatus* was performed using previously reported procedure (Lee et al. 1999) with slight modification. Briefly, *P. ostreatus* was pre-cultured on substrate mixture composed of 80% Douglas-fir sawdust and 20% rice bran packaged in heat-resistant bottle (1100 mL, φ75 mm) at 20 °C for 30 days. After mycelium was totally grown, 4 g of hyphae attached substrate was transferred into each buckwheat medium. The buckwheat medium contained milled unhulled buckwheat seeds in basal medium containing 66.7% (w/w) poplar saw dust, 16.7% (w/w) cotton-seed meal, and 16.7% (w/w) beet pulp. Moisture content was adjusted to 65% (w/w) before sterilization. Each milled buckwheat was mixed with basal medium at 20% (w/w) of nutritious substrate. The following media were prepared: basal (G) medium, common buckwheat (CB) medium, fermented common buckwheat (FCB) medium, tartary buckwheat (TB) medium, and fermented Tartary buckwheat (FTB) medium (Table 1). All media (620 g) were packed in heat-resistant bottle and sterilized serially at 100 °C for 30 min and 121 °C for 90 min. After sterilization, each medium was cooled down to room temperature. Pre-cultured *P. ostreatus* (4 g of wet weight) was then inoculated to each medium and incubated at 20 °C under 65% relative humidity in a dark room. Carbon dioxide was controlled to be 3000 to 5000 ppm to induce balanced shape of pileus and stipe (Sánchez 2010). After 30 days of incubation with mycelium to induce fruit body, old spawn was removed and temperature was maintained at 15 °C. Relative humidity and carbon dioxide were maintained over 90% and 500–3000 ppm, respectively. Each bottle possessed 0.056 m^3^ of space in the incubation room. On the 8th day after inducing fruit body, fruit bodies were harvested and 10 fruit bodies of each group were randomly selected. These fruit bodies were then lyophilized and stored at − 80 °C for further analysis.

**Table 1:** Composition and ratio of mushroom medium

Medium	Medium composition (g/bottle)							Medium weight per bottle (g)	Buckwheat proportion to medium (%, w/w)	Buckwheat proportion to total medium weight (%, w/w)
	Poplar saw dust	Cotton seed meal	Beet pulp	Buckwheat						
				CB	FCB	TB	FTB			
G	144.7	36.2	36.2	–	–	–	–	620	0	0
CB	135.3	33.8	33.8	14.0	–	–	–	620	20.74	2.26
FCB	135.3	33.8	33.8	–	14.0	–	–	620	20.74	2.26
TB	135.3	33.8	33.8	–	–	14.0	–	620	20.74	2.26
FTB	135.3	33.8	33.8	–	–	–	14.0	620	20.74	2.26

*G* basal medium, *CB* common buckwheat, *FCB* fermented common buckwheat, *TB* tartary buckwheat, *FTB* fermented tartary buckwheat

### Mushroom morphological characteristics

Morphological characteristics such as fruit body weight, pileus diameter, and stipe length were determined. Halogen lamb analyzer MB35 (Ohaus Inc., Parsippany, NJ, USA) was used to measure moisture content of fruit body. Color change of mushroom pileus was measured with Hunter’s color value (L* (ligh vs dark), a* (red vs green), b* (yellow vs blue)) with a colorimeter (Konica Minolta, Tokyo, Japan). Total size index (TSI) representing comprehensive morphological size was each morphological value. Each value was obtained by multiplication of weight (g), pileus diameter (cm), stipe length (cm), and stipe thickness (cm). Each obtained value was then rescaled (divided by 100) and its unit was omitted using the following equation:

$${\text{Total Size Index }} = \frac{(Mushroom \; weight \times Pileus \; diameter \times Stipe \; length \times Stipe \; thickness)}{100}$$

### Liquid chromatography analyses of mushroom components

l-carnitine content in buckwheat and mushroom was analyzed with liquid chromatography–electrospray ionization–tandem mass spectrometry (LC–ESI–MS) (Park et al. 2017). Each sample (100 mg) was extracted with water (1 mL) and centrifuged at 13,500×*g* for 10 min. The supernatant was diluted tenfold with acetonitrile. It was centrifuged again at 13,500×*g* for 10 min. The supernatant was filtered using 0.2 µm pore size syringe filter (Sartorius, Germany). For quercetin and rutin analysis, each sample (100 mg) was extracted with 70% ethanol (1 mL) followed by centrifugation and filtration as described above.

The filtrate (1 µL) was used for component analysis as follows. l-carnitine analysis was performed on an Acquity UPLC system equipped with ESI–MS and BEH 1.7 μm HILIC column (2.1 mm × 150 mm, Waters, USA). Mobile phase A was 15 mM ammonium formate with 0.1% (v/v) formic acid. Mobile phase B was acetonitrile with 0.1% (v/v) formic acid. Flow rate was set at 0.4 mL/min. Sample manager temperature was sustained at 20 °C while column temperature was maintained at 40 °C. Mobile phase A was sustained at 10% for initial 3 min, 30% for the next 2 min, 60% for 1 min, and 10% for the last 4 min. Each compound was recorded and quantified at specific ion mass [M + H^+^]. ESI–MS conditions were: ion mode, positive; capillary voltage, + 1.5 kV; cone voltage, − 10 V; and single ion recording, 162 g/mol. Quercetin and rutin were separated using Kromasil 1.8 μm C18 UHPLC column (2.1 mm × 50 mm, Kromasil, Bohus, Sweden). Flow rate was set at 0.3 mL/min. Mobile phase A was 0.1% (v/v) formic acid while mobile phase B was acetonitrile with 0.1% (v/v) formic acid. Temperature was set the same as described above. Mobile phase B was gradually increased from 30 to 100% in the initial 5 min. It was then decreased back to 30% in the last 3 min. ESI–MS conditions were: capillary voltage, + 1.5 kV; cone voltage, + 25 V for rutin and − 10 V for quercetin; single ion recording, 609.5 g/mol for quercetin and 303.2 g/mol for rutin. All the samples, quercetin and rutin standards kept in amber glass vial, which blocked penetration of light.

The calibration curve was prepared by the external standard method, with l-carnitine standard ranging from 0.0125 to 0.5 μg/mL and quercetin ranging from 0.05 to 2 μg/mL and rutin ranging 0.1 to 5 μg/mL. Each standard curves showed over 0.99 coefficient of determination (R^2^ > 0.99).

### Preparation of mushroom fruit body extract

Powdered mushroom fruit body (1 g) of each treatment (G, CB, FCB, TB, and FTB medium) was extracted with ethanol (10 mL) at 20 °C (200 rpm) for 24 h. The mixture was then centrifuged at 3500×*g* for 10 min. The supernatant was filtered using Whatman paper filter No. 1 (Whatman, Piscataway, NJ, USA). The filtrate was evaporated at 45 °C for 1 h and lyophilized using a freeze dryer (Eyela, Tokyo, Japan). The yield of extracted mushroom by ethanol was 17.2% (w/w). The extracted mushroom powder by ethanol was then re-dissolved in dimethyl sulfoxide (DMSO) solution (10 mg/mL) and used for in vitro cell cytotoxicity and antioxidant assays.

### Antioxidant activity of mushroom fruit body extract

Antioxidant activity of mushroom ethanol extract was evaluated using 2,2-diphenyl-1-picrylhydrazyl (DPPH) radical scavenging method described previously (Nguyen et al. 2017) with slight modification. Mushroom powder was extracted by ethanol, then ethanol was removed by evaporation and lyophilized at − 10 to 0 °C under 10 Pa (Eyela FD-550, Tokyo Rikakikai Co., Tokyo, Japan). The extracted mushroom powder was dissolved in DMSO and mixed with 100 µM of DPPH reagent dissolved in ethanol. The final concentration of mushroom extract ranged from 0.01 to 1 mg/mL. The mixture was incubated at room temperature for 30 min. The absorbance of each mixture was obtained at wavelength of 517 nm on a microplate. DMSO solution was used as negative control. Trolox was used as a positive control.

The relative radical scavenging activity (SC) was obtained with the following equation (Choi et al. 2018a, b):

$${\text{SC }}\left( {\text{\% }} \right) = \frac{{\left( {Abs \; of \; negative \; control - Abs \; of \; the \; sample} \right)}}{Abs \; of \; negative \; control} \times 100$$

Results were expressed as mean ± standard error of the mean (SEM). All analyses were carried out in triplicates.

### Cell viability assay of oyster mushroom ethanol extract

Murine macrophage cell line Raw 264.7 cells (Raw 264.7 cells) were grown in Dulbecco’s modified Eagle’s medium supplemented with 10% fetal bovine serum (FBS, GenDEPOT, Barker TX, USA), 100 unit/mL penicillin (GenDEPOT, Barker TX, USA), and 100 μg/mL streptomycin (GenDEPOT, Barker TX, USA) at 37 °C under 5% CO_2_ (Choi et al. 2018a, b; Maxwell et al. 2017). Extracted oyster mushroom powder was prepared in DMSO solution (10 mg/mL). RAW264.7 cells were seeded into 96-well cell culture plate at density of 2 × 10^4^ cells/well and incubated at 37 °C for 24 h in a humidified atmosphere containing 5% CO_2_. After discarding the culture medium, sample was then diluted with medium (1.17 to 1.2 mg/mL) and added to each well followed by incubation at 37 °C for 24 h. To evaluate cytotoxicity, WST-1 (Water soluble tetrazolium salt) assay was performed using EZ-cytox kit (Daeil Lab service, Seoul, Korea).

### Statistical analysis

All data were obtained after repeating the experiment three times except for morphological analysis. Morphological characteristics were obtained from 10 randomly selected samples. Mean value was given with standard error of the mean (SEM). The significant differences between groups were determined by Tukey’s HSD (Honest significant difference) methods (*P *< 0.05 or *P *< 0.01). Statistical analysis was performed using SPSS version 23.0 for Windows (SPSS Inc., Chicago, IL, USA).

## Results

### Levels of l-carnitine and phenolic compounds in fermented buckwheat

One kg of CB and TB contained 11.3 mg and 6.2 mg of l-carnitine, respectively (Table 2). After *R. oligosporus* fermentation, the amounts of l-carnitine in both buckwheats were increased (from 11.3 mg to 26.2 mg in 1 kg of FCB and from 6.2 mg to 38.4 mg in 1 kg of FTB). The increasing rate of l-carnitine was higher after TB fermentation (619.4%) than that after CB fermentation (231.8%). Contents of total phenol and flavonoids in fermented buckwheat were higher than those of non-fermented buckwheat. However, content of quercetin decreased after fermentation in both CB and TB. On the contrary, rutin content in TB increased from 3923.2 to 5148.1 mg/kg after fermentation.

**Table 2:** l-carnitine, total phenolic content, quercetin and rutin concentration in buckwheat after *R. oligosporus* fermentation

Buckwheat	l-carnitine (mg/kg)	Total phenolic content (mg GAE/kg)	Quercetin (mg/kg)	Rutin (mg/kg)
CB	11.3 ± 0.4	1470.1 ± 57.9	19.3 ± 0.4	75.9 ± 4.5
FCB	26.2 ± 0.6**	3466.8 ± 102.6**	14.7 ± 0.8**	71.4 ± 2.0
TB	6.2 ± 0.1	5372.3 ± 114.5	3155.6 ± 68.3	3923.2 ± 90.7
FTB	38.4 ± 0.5**	7279.3 ± 176.2**	2619.0 ± 89.7**	5148.1 ± 188.5**

*CB* common buckwheat, *FCB* fermented common buckwheat, *TB* tartary buckwheat, *FTB* fermented tartary buckwheat. TPC is expressed by garlic acid equivalent (GAE). Each mean value was written with standard error of the mean (SEM). For each parameter, asterisk star (* or **) means significantly different (*p* < 0.05 or *p* < 0.01) from non-fermented buckwheat

### Levels of l-carnitine in oyster mushroom in all samples

Amounts of l-carnitine in 1 kg of dried oyster mushroom grown on different media (CB, FCB, TB, and FTB medium) were compared. Results are shown in Table 3. When oyster mushroom was grown on FCB medium, the amount of l-carnitine was significantly (*P *< 0.01) increased (by 22.3%) compared to that grown on G medium. However, oyster mushroom grown on FTB medium had smaller increase in the amount of l-carnitine (by 12.9%) compared to that grown on G medium.

**Table 3:** L-carnitine content in oyster mushroom grown on various buckwheat media

Mushroom medium material	Mean (mg/kg)	Increase rate (%)	Mushroom medium material	Mean (mg/kg)	Increase rate (%)
G	164.5 ± 4.9				
CB	186.3 ± 8.1	13.3	TB	180.8 ± 6.2	9.9
FCB	201.2 ± 3.6**	22.3	FTB	185.7 ± 4.9	12.9

*G* basal medium, *CB* common buckwheat, *FCB* fermented common buckwheat, *TB* tartary buckwheat, *FTB* fermented tartary buckwheat. Each mean value was written with standard error of the mean (SEM). For each parameter, asterisk star (* or **) means significantly different (*p* < 0.05 or *p* < 0.01) from basal medium (G)

### Morphological characteristics of mushroom grown on buckwheat media

Mushroom weight and moisture content were similar to each other for all samples. They were not significantly (*P* > 0.01) different compared to those of mushroom grown on G medium (Table 4). Mushroom size was represented by total size index (TSI). TSI of oyster mushroom grown on FCB medium was significantly (*P* < 0.01) increased compared to that grown on G medium. However, other size parameters such as pileus diameter, stipe length, and stipe thickness were not significantly changed except pileus diameter in FCB medium and stipe length in CB medium.

**Table 4:** Morphological characteristics of oyster mushroom

medium	Cultivation number	Sampling number	Formation rate (%)	Mushroom weight (g/bunch)	Moisture (%)	Pileus diameter (cm)	Stipe length (cm)	Stipe thickness (cm)	Color			Total size index^†^
									L*	a*	b*	
G	48	10	97.9	170.6 ± 7.8	89.7 ± 0.3	3.0 ± 0.1	8.1 ± 0.4	1.2 ± 0.0	41.8 ± 0.9	2.6 ± 0.1	4.1 ± 0.2	52.1 ± 6.3
CB	32	10	96.9	187.3 ± 7.8	88.1 ± 0.5	3.0 ± 0.2	10.3 ± 0.5**	1.3 ± 0.1	50.1 ± 1.6**	3.6 ± 0.2**	6.3 ± 0.5**	78.9 ± 11.5
FCB	32	10	93.8	181.4 ± 5.3	88.3 ± 0.4	4.0 ± 0.2**	9.5 ± 0.6	1.4 ± 0.0	50.6 ± 0.9**	3.6 ± 0.1**	6.5 ± 0.4**	100.7 ± 11.7*
TB	32	10	96.9	180.9 ± 10.5	89.7 ± 0.2	3.2 ± 0.2	10.0 ± 0.5	1.3 ± 0.0	48.2 ± 0.8**	3.5 ± 0.1**	5.8 ± 0.4	80.9 ± 11.6
FTB	32	10	96.9	182.6 ± 9.0	89.5 ± 0.2	3.2 ± 0.2	10.1 ± 0.3	1.3 ± 0.0	49.2 ± 0.8**	3.6 ± 0.1**	6.6 ± 0.4**	82.1 ± 11.5

*G* basal medium, *CB* common buckwheat, *FCB* fermented common buckwheat, *TB* tartary buckwheat, *FTB* fermented tartary buckwheat. For each parameter, values in the same column with asterisk star (* or **) are significantly different (*p* < 0.05 or *p* < 0.01) from basal medium (G). Each mean value was written with standard error of the mean (SEM). ^†^ ${\text{Total size index}}\, = \,(Mushroom weight \times Pileus diameter \times Stipe length \times Stipe thickness)/100$

### Color index of mushroom grown on buckwheat containing medium

By adding buckwheat into the media, Lightness (L*) values of all mushroom samples were significantly increased (*P* < 0.01) compared to those grown on G medium (Table 4). Yellowness (b*) values were also increased for all mushrooms grown on media added with buckwheat. However, yellowness values of mushrooms grown on TB medium were not significantly (*P* > 0.01) different from those of mushrooms grown on G medium.

### Antioxidant activities of ethanol extracts of mushrooms

**Fig. 1:**
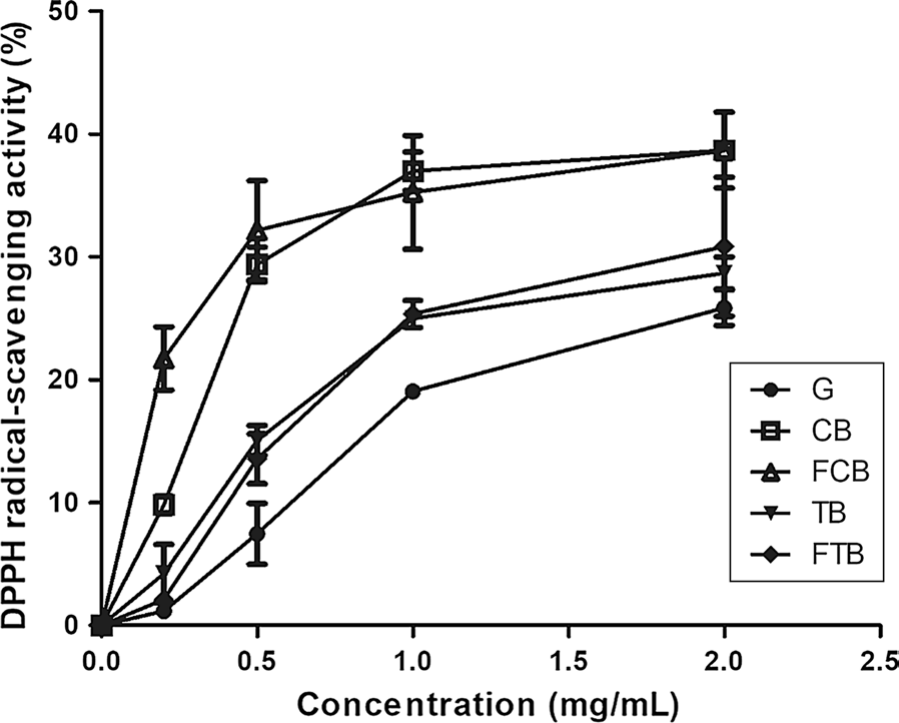
DPPH radical scavenging activities of ethanol extract of oyster mushroom fruit body. *G* basal medium, *CB* common buckwheat medium, *FCB* fermented common buckwheat medium, *TB* tartary buckwheat medium, *FTB* Fermented Tartary buckwheat medium. Each mean value was written with standard error of the mean (SEM)

Antioxidant effects of mushroom ethanol extracts against DPPH were evaluated at concentration ranging from 0.2 to 1.5 mg/mL. Results are shown in Fig. 1. Radical scavenging activities of all samples were increased in a concentration-dependent manner. At final concentration of 1.5 mg/mL, radical scavenging activity of oyster mushroom ethanol extract ranged from 25.9 to 38.7%. At this concentration, ethanol extract of mushroom grown on G medium was found to be 25.9%. Ethanol extracts of mushrooms grown on CB and FCB medium showed the same scavenging activity (both at 38.7%). Those of mushrooms grown on TB and FTB medium showed scavenging activities of 28.7 and 30.9%, respectively. Trolox used as positive control (Additional file 1: Figure S1).

### Cell cytotoxicity ethanol extract of oyster mushroom against Raw 264.7

**Fig. 2:**
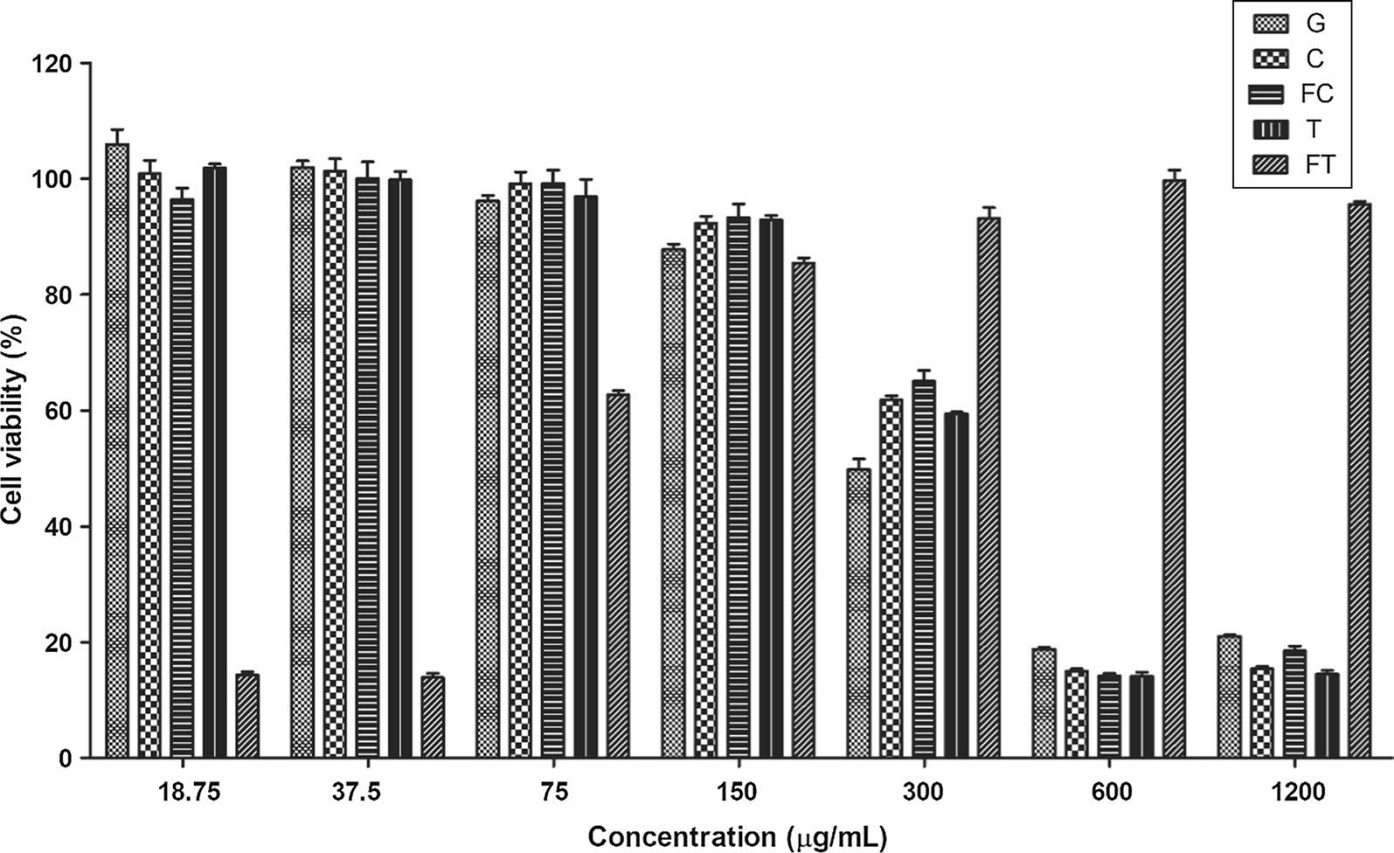
Cell cytotoxicity of ethanol extracted oyster mushroom against Raw 264.7 cells. Ethanol extracted oyster mushroom grown up on *G* basal medium, *CB* common buckwheat medium, *FCB* fermented common buckwheat medium, *TB* tartary buckwheat medium, *FTB* fermented tartary buckwheat medium against Raw 264.7 cells

Cytotoxicity of ethanol extract of mushroom fruit body to Raw 264.7 cells was evaluated at a final concentration ranging from 18.75 to 1200 μg/mL (Fig. 2). At a final concentration of 0 to 75 μg/mL, viability of Raw 264.7 cells was above 90% after treatment with all ethanol extract samples. Up to 150 μg/mL, the viabilities of Raw 264.7 cells treated with ethanol extracts of all mushroom samples were not significantly (*P *> 0.05) different from those of control cells without ethanol extract treatment. At final concentration of 300 μg/mL, viabilities of cells treated with ethanol extract of oyster mushroom grown on buckwheat medium ranged from 58.9 to 67.8%.

## Discussion

In this study, the amounts of l-carnitine in FCB and FTB by *R. oligosporus* were increased 2.3 times and 6.2 times compared to CB and TB (Table 3). l-carnitine is synthesized from lysine and methionine (Bremer 1983), thus the synthesis of l-carnitine depends on the amount of lysine and methionine in buckwheat. The protein, lysine, methionine contents (25.3% (w/w), 58.8 g/kg, and 13.3 g/kg) in TB bran were higher than that in CB bran (21.6% (w/w), 54.7 g/kg, and 1.09 g/kg) (Bonafaccia et al. 2003). The fermentation process of buckwheat by *R. oligosporus* can increase the amino acid content in buckwheat, thus the l-carnitine contents in FTB are higher than that of FCB. The total phenolic contents in FCB and FTB were increased 2.4 times and 1.4 times compared to CB and TB. Among phenolic compounds in buckwheat, rutin and quercetin were considered as major bioactive compounds in buckwheat. There is a wide variation of rutin content in buckwheat seed depending on the species, variety, and the environmental conditions under which they are produced (Jiang et al. 2007). Therefore, rutin and quercetin were selected in buckwheat and fermented buckwheat. Although quercetin contents in fermented buckwheat were decreased compared to non-fermented buckwheat, rutin contents in FTB was increased 1.3 times compared to TB. McCue and Shetty (2003) reported that α-amylase and endogenous carbohydrate-cleaving enzymes produced from *R. oligosporus* can generate polyphenols from carbohydrate-conjugated phenolic compounds during fermentation of buckwheat. In addition, *R. oligosporus* is a known strain to produce β-glucosidase, β-glucuronidase and xylanase when it degrades the cell wall matrix (Huynh et al. 2014; Varzakas 1998). Thus, it probably metabolizes extracellular components with bioconversion of phenolic compounds by the fermentation that leads the cell-wall degrading enzymes to hydrolyze glycosidic bonds and produces unbound phenolics and aglycone forms. The fermentation processes releasing phenolic compounds from plant matrixes followed by the metabolic pathways of flavonoids: glycosylation, deglycosylation, ring cleavage, methylation, glucuronidation, and sulfate conjunction which are the ways of producing new bioactive compounds as well as increasing the total phenol contents and rutin contents in fermented buckwheat (Huynh et al. 2014).

Oyster mushroom grown on buckwheat and fermented buckwheat had higher l-carnitine contents than mushroom grown on normal medium (Table 3) and the l-carnitine contents in mushroom grown on FCB was similar to the amount of l-carnitine in pork muscle (Demarquoy et al. 2004). l-carnitine content in oyster mushroom grown on fermented buckwheat medium was higher than mushroom grown on non-fermented buckwheat medium. It could be explained on the grounds that *R. oligosporus* can synthesize various l-carnitine derivatives that might be supplied to mycelia of oyster mushroom. Further study is needed to obtain clearer explanation. One of the reasons for the higher antioxidant activities of ethanol extracts from mushrooms grown on CB, TB, FCB and FTB medium compared to those of mushroom grown on G medium (Fig. 1) is the presence of l-carnitine which is known to possess antioxidant activity (Holasova et al. 2002).

The Raw 264.7 cell viability in the presence of ethanol extract from oyster mushroom grown on buckwheat medium was higher than that of oyster mushroom grown on G medium. It has been reported that l-carnitine can reduce oxidative stress in in vitro cell culture of Raw 264.7 cells and HK-2 cells (Koc et al. 2011; Ye et al. 2010). l-carnitine is also known to have anti-inflammatory activity because it can suppress inducible nitric oxide synthase (iNOS) that produces nitric oxide at transcriptional level (Koc et al. 2011). Therefore, the higher in Raw 264.7 cell viability might be due to its higher content of l-carnitine. However, the pattern of l-carnitine concentration in mushroom was not exactly the same as that of mushroom extracts cytotoxicity against Raw 264.7 cells. Therefore, l-carnitine might not be the only factor involved in the less cytotoxicity of ethanol extract from oyster mushroom grown on buckwheat media. Further studies are needed to understand the underlying mechanisms.

## Additional file

**Additional file 1: Figure S1.** DPPH radical scavenging activity of trolox.

## Abbreviations

*P. ostreatus*: *Pleurotus ostreatus*
*R. oligosporus*: *Rhizopus microspores* var. *oligosporus*
TPC: total phenol content
TFC: total flavonoids content
G: basal
CB: common buckwheat
FCB: fermented common buckwheat
TB: tartary buckwheat
FTB: fermented tartary buckwheat
TSI: total size index
LC–ESI–MS: liquid chromatography–electrospray ionization–tandem mass spectrometry
DMSO: dimethyl sulfoxide
DPPH: 2,2-diphenyl-1-picrylhydrazyl
SC: scavenging activity
SEM: standard error of the mean
iNOS: nitric oxide synthase
