# Supplementary material for: The use of fermented buckwheat to produce l-carnitine enriched oyster mushroom
Source: AMB Express. 2018 Aug 27;8:138. doi: 10.1186/s13568-018-0664-6 (PMC6111021; doi:10.1186/s13568-018-0664-6)
Supplement: Supplementary file 1 — Additional file 1: Figure S1. DPPH radical scavenging activity of trolox. [file 13568_2018_664_MOESM1_ESM.docx]

**Additional file**

**The Use of fermented buckwheat to produce L-carnitine enriched oyster mushroom**

Tae-kyung Lee^1^, Thi Thanh Hanh Nguyen^2^, Namhyeon Park^1^, So-Hyung Kwak^1^, Jeesoo Kim^1^, Shina Jin^1^, Gyu-Min Son^1^, Jaewon Hur^1^, Jong-In Choi^3^, Doman Kim^1,2*^

^1^Graduate School of International Agricultural Technology and Center for Food and Bioconvergence, Seoul National University, Pyeongchang 232-916, Korea. Email: vluetk@snu.ac.kr (T-K Lee); sayparknh@naver.com (N.H. Park); shkwak16@snu.ac.kr (S-H Kwak); j_k084@snu.ac.kr (JS Kim); jsn0705@snu.ac.kr (SN Jin); handson@snu.ac.kr (G-M Son); willbe17@snu.ac.kr (JW Hur); kimdm@snu.ac.kr (D Kim).

^2^The Institute of Food Industrialization, Institutes of Green Bio Science &Technology, Seoul National University, Pyeongchang 232-916, Korea. Email: hara2910@snu.ac.kr (TTH Nguyen);

^3^Mushroom Research Institute, GARES, Gyeonggi Gwang-ju 464-870, Korea. Email: cji190@gg.go.kr (J-I Choi)

^*^ Corresponding author.

E-mail: kimdm@snu.ac.kr Tel: +82-33-339-5720; Fax: +82-33-339-5716

**
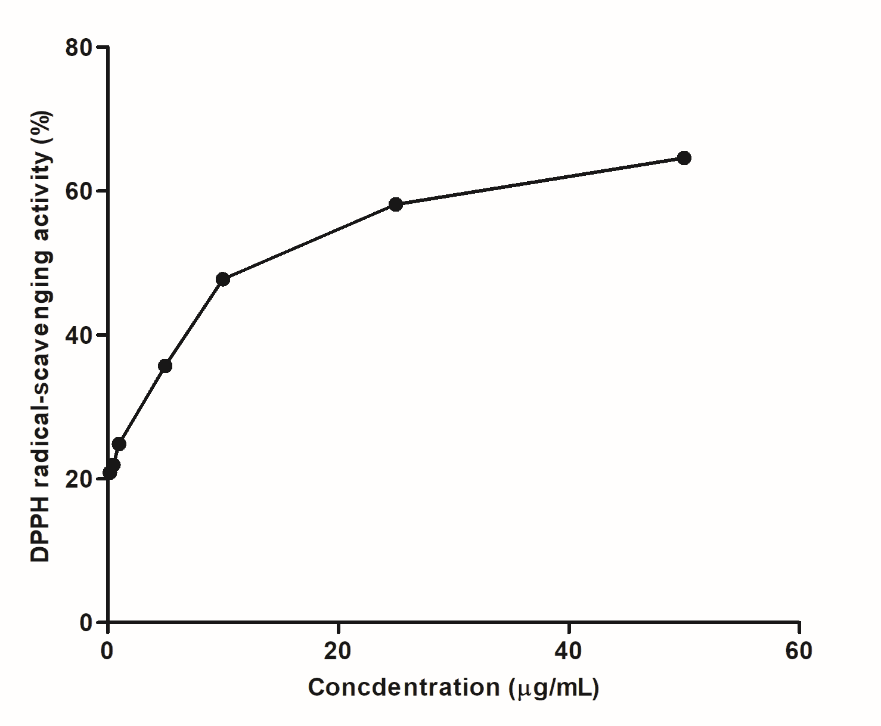
**

**Fig. S1.** DPPH radical scavenging activity of trolox.
